# Plant breeding advancements with “CRISPR-Cas” genome editing technologies will assist future food security

**DOI:** 10.3389/fpls.2023.1133036

**Published:** 2023-03-13

**Authors:** M. AHMAD

**Affiliations:** ^1^Department of Plant Sciences, University of Nebraska, Lincoln, NE, United States; ^2^Department of Genetics and Plant Breeding, Sheri-e-Kashmir University of Agricultural Sciences and Technology-Kashmir, Srinagar, India

**Keywords:** genome editing, Food security, breeding techniques, CRISPR-Cas, Future development, breeding techniques

## Abstract

Genome editing techniques are being used to modify plant breeding, which might increase food production sustainably by 2050. A product made feasible by genome editing is becoming better known, because of looser regulation and widespread acceptance. The world’s population and food supply would never have increased proportionally under current farming practices. The development of plants and food production has been greatly impacted by global warming and climate change. Therefore, minimizing these effects is crucial for agricultural production that is sustainable. Crops are becoming more resilient to abiotic stress because of sophisticated agricultural practices and a better understanding of the abiotic stress response mechanism. Both conventional and molecular breeding techniques have been used to create viable crop types both processes are time-consuming. Recently, plant breeders have shown an interest in genome editing approaches for genetic manipulation that use clustered regularly interspaced short palindromic repeats (CRISPR/Cas9). To ensure the security of the food supply in the future, plant kinds with desired traits must be developed. A completely new era in plant breeding has begun because of the revolution in genome editing techniques based on the CRISPR/CRISPR-associated nuclease (Cas9) systems. All plants may effectively target a particular gene or group of loci using Cas9 and single-guide RNA (sgRNA). CRISPR/Cas9 can thereby save time and labor compared to conventional breeding methods. An easy, quick, and efficient method for directly altering the genetic sequences in cells is with the CRISPR and Cas9 systems. The CRISPR-Cas9 system, which was developed from components of the earliest known bacterial immune system, allows for targeted gene breakage and gene editing in a variety of cells/RNA sequences to guide endonuclease cleavage specificity in the CRISPR-Cas9 system. Editing can be directed to practically any genomic site by altering the guide RNA (gRNA) sequence and delivering it to a target cell along with the Cas9 endonuclease. We summarize recent CRISPR/Cas9 plant research findings, investigate potential applications in plant breeding, and make predictions about likely future breakthroughs and approaches to food security through 2050.

## 1 Challenges in 21st-century food systems

By 2050, the environmental, agricultural, and food difficulties of the twenty-first century will pose serious global problems (Okuzaki and Toriyama, 2004; Ma et al., 2017; Metje-Sprink et al., 2018). When the world’s population reaches 9.7 billion, there will be a 50% increase in demand for agricultural products over 2012 (FAO, 2017). The demand for high-quality food will be impacted by disparities in nutritional needs between young and old people, as well as consumption habits, occupations, and living arrangements between urban and rural populations. Because of growing food prices, changes in agricultural output, and rising wealth in low- and middle-income nations, people will consume more meat, fruits, and vegetables compared to grains (FAO, 2017). Despite expenditures in agricultural and technical advancements, crop growth has remained constant for the past three decades. Global staple crop yields have grown by a little over 1% annually on average since the 1990s, a substantially lower rate than in the 1960s (FAO, 2017). The need for productivity to accelerate due to factors like climate change, resource depletion, biodiversity loss, and the development of plant pests and diseases (FAO, 2017). Different locations, ecological zones, and production systems are anticipated to experience the effects of climate change to varying degrees and in various ways (FAO, 2017). Increased frequency of drought and flooding as well as rising unpredictability of precipitation may all harm yields. The fact that studies have demonstrated that when daytime temperatures reach a certain crop-specific level, warmer temperatures can stimulate crop development (FAO, 2017). Due to changes in temperature and moisture levels that might encourage the growth of infections, fungi, and insects, climate change may also make plants more vulnerable to pests and diseases (FAO, 2017). In addition to affecting climate change, food, and agricultural systems also significantly contribute to it. Agricultural emissions still make up to 20% of all global greenhouse gas (GHG) emissions, although emissions from forestry, agriculture, and other land uses have practically stabilized over the past 25 years (FAO, 2017). However, there are ways to mitigate the damage. Agricultural techniques and technology that boost food production while utilizing fewer “GHG-intensive” methods must be developed to combat climate change. According to the European Commission, this strategy will help ensure that Europeans have access to wholesome, affordably priced, and sustainably produced food. Additionally, it aims to combat climate change, safeguard the ecosystem and biodiversity, guarantee a just financial return along the food supply chain, and promote organic farming (Swaminathan, 2009; Verhaegen and De Smet, 2017; Van Vu et al., 2019; Schmidt et al., 2020b).

### 1.1 The farm-to-fork strategy’s particular goals are to:

By 2030, (FAO, 2017)

- Cut down on the use of chemical pesticides and the associated risks by 20%
- Cut back on the use of more dangerous pesticides by 50%; and
- Ensure that soil fertility is preserved, preventing at least 50% of nutrient loss

Even though thereby applying CRISPR-Cas technology in studies that seek to understand how genes function in complex biological processes and by using these techniques to introduce certain desired genetic mutations into a plant, genome editing can help to improve crop genetics in two ways. Genome editing is a technique used by scientists to discover candidate genes and genetic variants that affect desirable traits, better harness genetic diversity, and understand how genes function. Research using genome editing does not always result in crops that have been altered in the genome. It can also be employed to direct conventional breeding techniques. Plant breeders will use a variety of techniques depending on the problem they’re attempting to solve. Each tool has benefits in terms of effectiveness, speed, and accuracy. Older breeding tools might still be able to accomplish some breeding goals, albeit at the sacrifice of precision and speed. Conventional breeding typically requires nine to eleven years, depending on the type of crop, to produce a new variant that may be sold. The process of creating new fruit tree varieties through traditional breeding takes a lot longer. The possibility for faster time to market exists with genome editing (Wang Z et al., 2022; Wang L et al., 2022). Elite plant species can also obtain precise genetic modifications through genome editing while avoiding other undesirable genetic changes (Whelan et al., 2020; Kashojiya et al., 2022). The pipeline for genome-edited crops and other elements included in the table will be used to determine whether a genome-edited crop can increase the overall sustainability of the food chain. Numerous studies have examined and identified a wide range of uses for genome editing in plants (Menz et al., 2020; Osakabe et al., 2020).

Among these uses include

- Improved disease resistance to lessen the demand for pesticides, among others. Enhanced resistance to abiotic stress to reduce the impact of climate change on our food production
- Improved agronomic traits to boost productivity, enhance crop yields, and reduce pre-harvest losses
- Improved character attributes, strengthened qualities relating to health (Duan et al., 2021).

The most recent genomic technologies, particularly those based on CRISPR, which was just recently discovered, and/or regulatory ambiguity regarding these methods in many countries could be the causes of this. However, it is anticipated that a lot more applications will start to develop and reach the market in the future. Currently, two crops with altered genomes are commercially available: tomatoes rich in gamma-aminobutyric acid (GABA) in Japan and soybeans high in oleic acid in the United States. A nutrient-rich mustard leaf will be offered in the US in 2023. In the upcoming years, many more are probably going to be put on the market. Due to the higher proportion of oleic and lower proportion of linolenic fatty acids in high oleic soybeans, the oil has a higher thermal and oxidative stability (Nonaka et al., 2017; Metje-Sprink et al., 2018; Ahmad M., 2022).

## 2 Introduction

**Figure 1:**
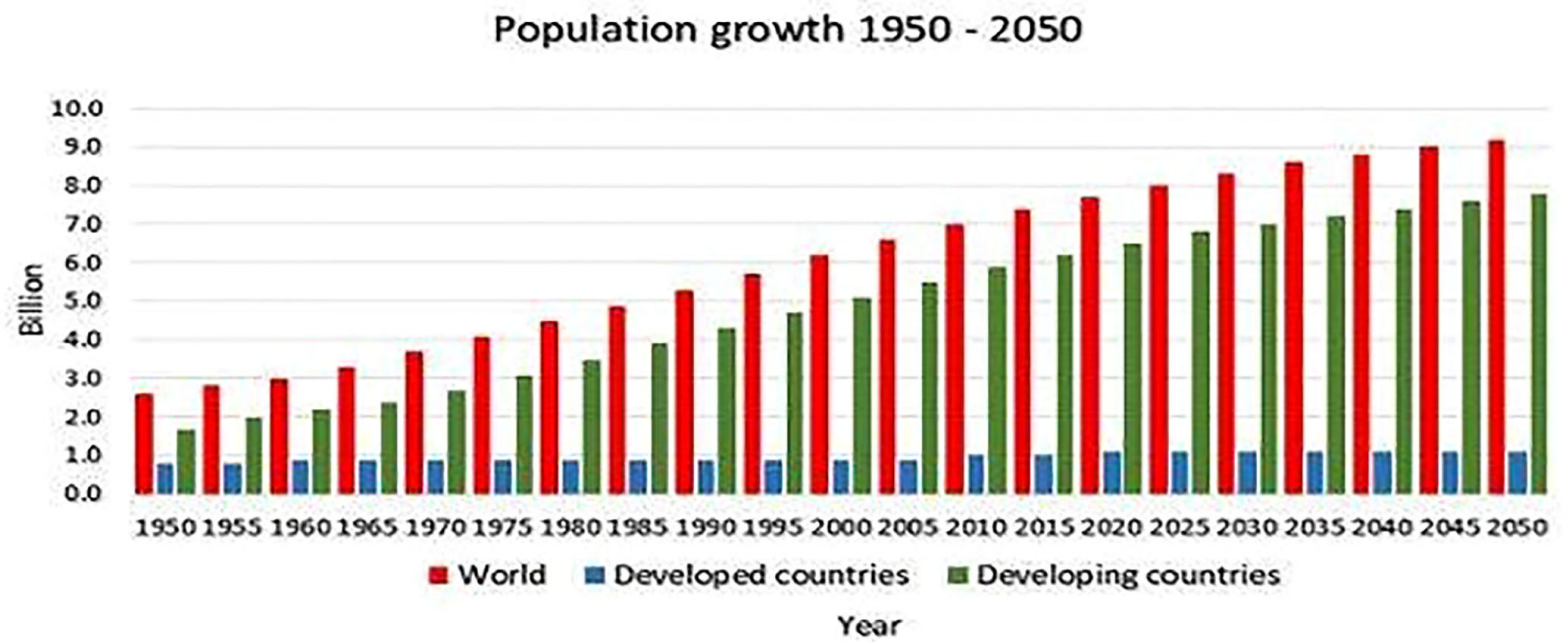
Population difference between developed and developing countries.

Due to the rise in global population and the need to address difficulties brought on by climate change, sustainable agriculture should be promoted. New crop varieties can be created by breeding techniques like annotation choice and traditional selection, but they take time. However, genome-editing technologies, particularly the CRISPR-(Cas-9) systems, are biotechnological tools for precise genetic change of crops that can cut breeding time while increasing diversity. This article provides a thorough explanation of the Cas9-CRISPR network with an emphasis on how it can be utilized to quickly create crop and plant varieties that are resistant to salinity, drought, and temperature stressors such as wheat, rice, tomato, soybean, oilseeds, potato, and maize (Laity et al., 2001; Puchta, 2005; Urnov et al., 2005; Scully et al., 2019; Rönspies et al., 2021; Ahmad M., 2022); To develop novel plant types that are resistant to abiotic stress or to apply to other underutilized crops for future food security, which is a major challenge up to 2050 in developing countries (**Figure 1**) the aforementioned crops contain crucial information on plant genome editing targets.

A growing number of fields, including plant science and crop breeding, characterization of gene function, and crop enhancement are turning to genetic modification as a cutting-edge and precise strategy of genetic modification. This includes editing to change a specific gene inside an organism’s genome (Anzalone et al., 2019; Scully et al., 2019; Yu et al., 2019; Hahn et al., 2020; Menz et al., 2020; Schmidt et al., 2020b; Ahmad M., 2022).

## 3 Why CRISPR-Cas GE better than ZFNs and TALENs genome editing?

Zinc-finger nucleases (ZFNs), transcription activator-like effector nucleases (TALENs), and the CRISPR/Cas system are the three primary categories of genome-editing tools. By causing DNA double-strand breaks, which promote error-prone non-homologous end joining (NHEJ) or homology-directed repair (HDR) at specified genomic loci, ZFNs and TALENs have been used to carry out a variety of genetic changes (Li et al., 2011; Miller et al., 2011; Jinek et al., 2012; Li et al., 2012; Gaj et al., 2013; Ahmad M., 2022). The DNA cleavage domain of the restriction enzyme FokI and zinc-finger-based DNA recognition modules make up the ZFN family of targeted drugs (Podevin et al., 2013; Pinto-Carbó et al., 2016; Verhaegen and De Smet, 2017; Ramkumar et al., 2022). Before joining together to cling to particular DNA sequences, each zinc finger locates and bonds to a nucleotide triplet. However, it took nine years from the discovery of ZFNs to the first ZFN-based plant genome editing due to the challenge of developing ZFNs with high sequence affinity and high off-target efficiency (Zetsche et al., 2015; Kim et al., 2016; Komor et al., 2016; Anzalone et al., 2019; Zhang et al., 2019; Lin et al., 2020; Ahmad M., 2022). The transcriptional activator-like effector (TALE) repeats are combined with the FokI restriction enzyme to produce TALENs (Hua et al., 2022; Kumar et al., 2022; Randall et al., 2022). Repetition variable residues (RVDs) and hypervariable amino acids at the 12th and 13th positions, such as adenine (A), cytosine (C), thymine (T), and guanine (G)/adenine (A), were identified using four distinct amino acid RVDs (NI, HD, NG/HG and NN), which form the core domain of TALE in this genome editing technology (Ahmad M., 2022; Hua et al., 2022; Kumar et al., 2022; Randall et al., 2022). The DNA identifiers link the network of amino acid repeats to the nucleotide sequence of the genome, enabling the construction of TALENs with specific sequence characteristics. Compared to ZAFN and TALN, the Cas9-CRISPR system is today a popular and effective gene-editing tool. CRISPR sequences can recognize foreign viral DNA and directly cleave Cas proteins as a part of the bacterial adaptive immune system (Zetsche et al., 2015; Kim et al., 2016; Komor et al., 2016). The Cas9 protein and synthesized single-guide RNA (sgRNA), which is produced by fusing CRISPR-RNA (crRNA) and transactivation RNA, are the main elements of the Cas9-CRISPR system (tracrRNA). Site-specific DNA double-strand breaks (DSBs), which are induced by the Cas9 protein under the control of sg-RNA, trigger DNA repair mechanisms. NGG for precision plant breeding and the research of gene function in plants have both made significant use of the technique. In the instance of Cas9-CRISPR, the target sequence was meant to be upstream of the protospacer-associated motif (PAM) (Verhaegen and De Smet, 2017; Fajrial et al., 2020; Menz et al., 2020; Vangheluwe et al., 2020; Kumar et al., 2022).

**Figure 2:**
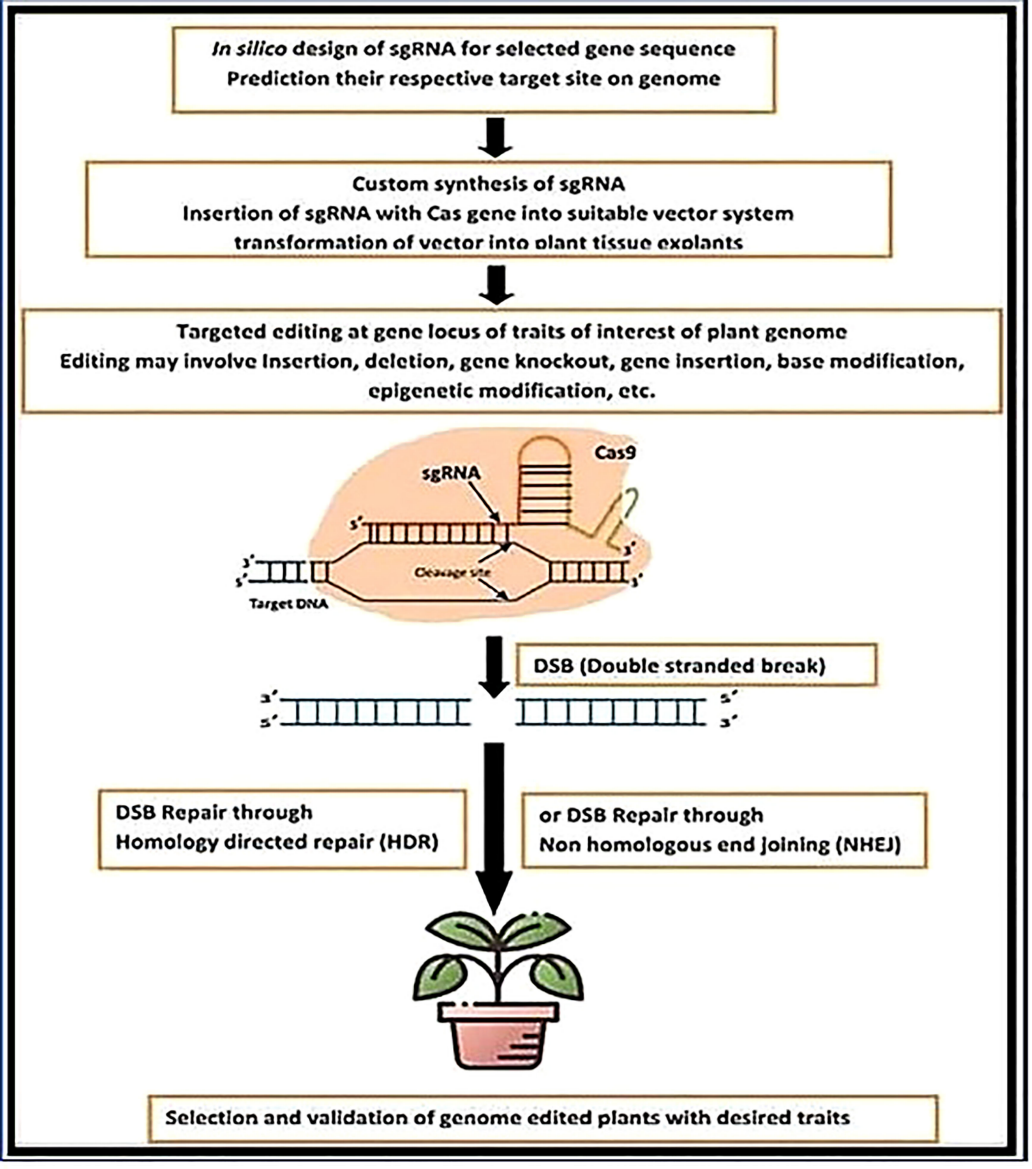
CRISPR-Cas genome editing methodology for developing genetically edited plants.

Despite the effectiveness of the Cas9-CRISPR system, other Cas-9 proteins have been discovered and used in genome editing technology. Cas12a/Cpf1, for example, is the most well-known Cas protein in addition to Cas9; it can distinguish between different PAM sequences and generate sticky ends rather than blunt ends, making HDR correction easier and leading to more precise editing (**Figure 2**). Some of the Cas enzyme subtypes include Cas3, Cas10, Cas12, Cas12b, Cas13a (Liu et al., 2020; Assou et al., 2021; Behr et al., 2021; Kumar et al., 2022), and CasF (Kumar et al., 2022). The possibilities available to different plant kinds may be increased by these various enzymes (Kumar et al., 2022). SpCas9 produced by Streptococcus pyogenes has been frequently employed (Anzalone et al., 2019; Liao et al., 2019; Zhang et al., 2019; Zhang et al., 2020; Lim et al., 2022). SaCas9, a Cas9 protein produced from Staphylococcus aureus, is smaller and better suited for intracellular trafficking than SpCas9 (Verhaegen and De Smet, 2017; Voss-Fels et al., 2019; Menz et al., 2020; Collias and Beisel 2021). Other Cas proteins, such as *StCas9* from Streptococcus thermophilus, NmCas9 from Neisseria meningitides, and FnCpf1 from Francisella novicida have also been discovered that target a variety of (PAM) sequences (Han and Kim, 2019; Akella et al., 2022; He et al., 2022; Kumar et al., 2022; Randall et al., 2022).

Both prokaryotes and eukaryotes have made significant use of the Cas9-CRISPR system for genome editing by extensive base pairing between the target DNA and a single guide RNA (sgRNA). The target region must also be surrounded by a short protospacer adjacent motif (PAM) sequence before the Cas9 recognizes and cleaves the target DNA (Chen et al., 2022; Chen S et al., 2021, Chen M et al., 2021; Hajian et al., 2019; Ganie et al., 2021; Gardiner et al., 2022; Gehrke et al., 2022). A sgRNA may cause unanticipated, off-target changes in the cell when it recognizes a non-target region that is almost identical to the target region and has few base mismatches. To minimize off-target changes, pairs of sgRNAs with similar target sites in opposite strands were used in conjunction with the mutant Cas9 (nickase). The mutant form only has one active domain, either HNH or RuvC, which results in a double-strand break and nicks one strand at the target site Gao, 2021; Kumar et al., 2022. As a result, flexible targeting or mining for PAM-free nucleases is becoming more and more popular. A PAM-free nuclease has the advantage of being able to target any sequence. As a result, picking places with high on-target activity and low off-target activity is easier (Raman, 2017; Bally et al., 2018; Chakrabarti et al., 2019; Gao, 2021).

This technique’s efficacy hinges on the efficient transcription, precise transport, and maximum activity of sgRNA and Cas9 at the desired target location with the fewest “off-target effects,” or unwanted changes to the genome (Aslam et al., 2021). The practice of synthetic biology has changed as a result of contemporary genome editing technologies. Since the advent of molecular biology in the middle of the 1990s, technologies like CRISPR/Cas-based gene editing have provided several methods for altering genomes and assisting plant scientists in realizing their aspirations. In the past 7 years, this technique has been widely used in almost every aspect of life, including agriculture, medicine, and the environment. After successfully using gene editing to change basic traits, scientists are now working on more ambitious genomics projects to solve the challenges of food security in the wake of the growing population and climate change risks (Ahmad et al., 2021; Aslam et al., 2021; Khan et al., 2021; Ashraf et al., 2022; Wang Z et al., 2022).

### 3.1 CRISPR/Cas9 System’s delivery mechanism

**Figure 3:**
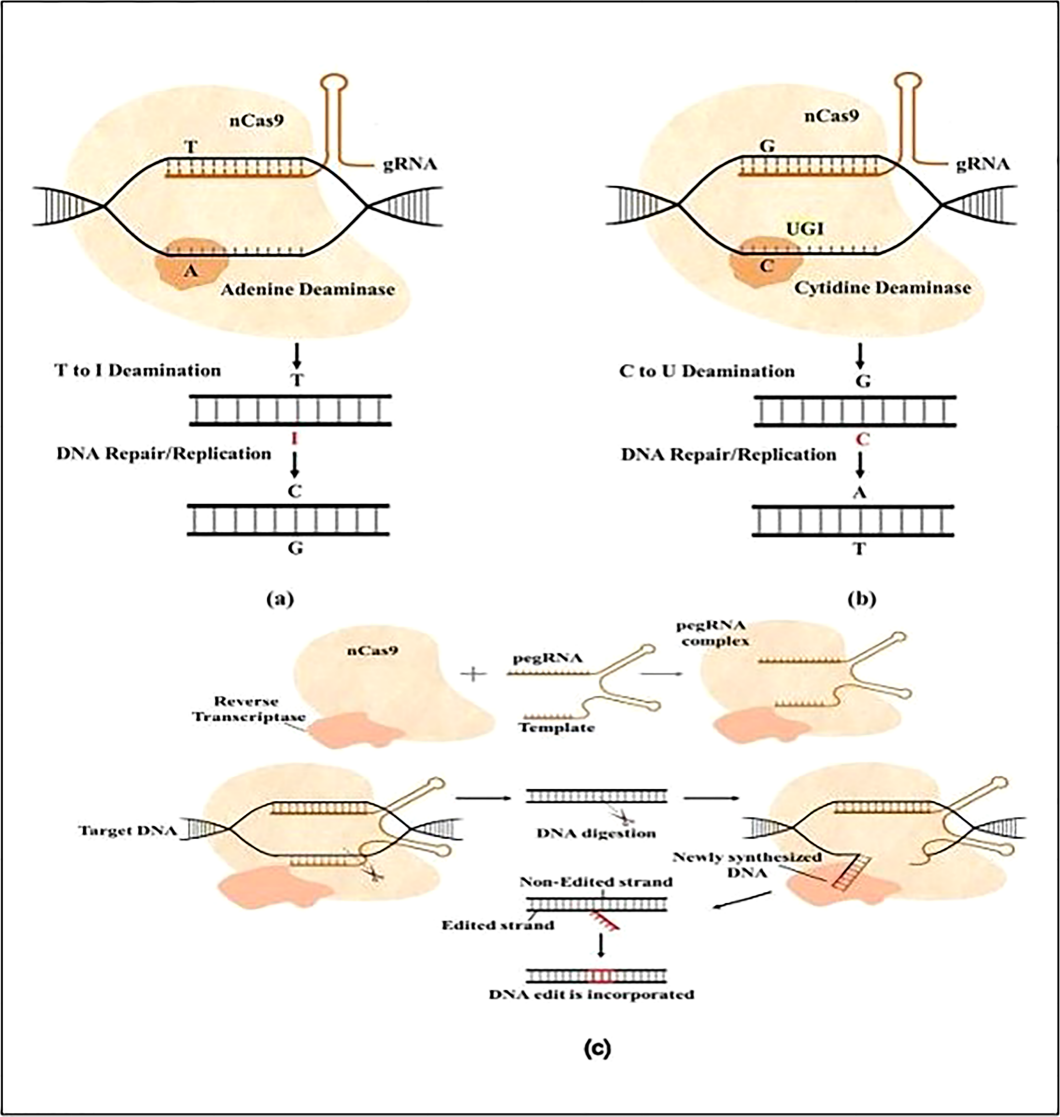
Schematically shown base modification with Nickase Cas9(nCas9), **(A)** The ABE system catalyzes the conversion of adenine into guanine *via* nCas9 and adenine deaminase. Adenine is deaminated by ABE to inosine (I), changing T-A to T-I. Repair machinery interprets I as G and repairs T-I as C-G; **(B)** the CBE system uses nCas9 and cytidine deaminase to catalyze the conversion of cytosine to uridine. UGI (uracil glycosylase inhibitor) inhibits U: G mismatch from being repaired back to C: G, causing U to ultimately change into T; **(C)** Prime editing is illustrated in a schematic by combining nCas9 with reverse transcriptase and a prime editing guide RNA (pegRNA). The reverse transcriptase may carry out a variety of transitions, insertions, and deletions, while prime editing mechanisms edit DNA without resulting in DSBs (Ahmad et al., 2021).

Researchers also found that there were both pre-existing humoral (anti-Cas-9 antibody) and cellular (anti- Immune reactions to the Cas-9 protein in healthy persons (Cas-9 T cells). Therefore, one of the biggest hurdles in the system’s clinical trial is currently figuring out how to identify and lessen the Cas-9 protein’s immunogenicity (Charlesworth et al., 2020). Worth For CRISPR/Cas-9 gene editing to work, the components must be delivered into the cell safely and effectively (**Figures 2**, **3**).

Physical, chemical, and viral vectors are the current three ways to introduce the CRISPR/Cas-9 complex into cells. Ex vivo CRISPR/Cas-9-based gene editing therapy is better suited to non-viral (physical and chemical) approaches. CRISPR/Cas-9 can be delivered physically using electroporation, microinjection, hydrodynamic injection, and other techniques (Huang and Puchta (2021); Ogata et al., 2020; Huang et al., 2022; Omukai et al., 2022; Tanwar et al., 2022). The CRISPR/Cas-9 complex can enter the cytoplasm of the target cell using electroporation, which uses a high electric field to temporarily increase the permeability of the cell membrane. The fundamental drawback of this approach is that it results in considerable cell death (Zhang et al., 2020). (For quick gene editing of a single cell, microinjection involves injecting the CRISPR/Cas-9 complex directly into cells at the tiny level. However, this approach also has several drawbacks including cell damage, which is technically difficult and is only appropriate for a small number of cells (Fajrial et al., 2020). The hydrodynamic injection is a quick, high-pressure liquid infusion into an animal’s bloodstream. Mice tail veins are typically used for this procedure. Despite being easy, quick, effective, and versatile, this technology has not yet been applied in clinical settings due to potential risks. Lipid- and polymer-based nanoparticles are used in the chemical delivery of CRISPR/Cas-9. When creating lipid nanoparticles or liposomes in aqueous solutions, chemicals based on Lipofectamine are used to create spherical structures made of the lipid bilayer membrane. The fusing of the complex across the cell membrane is made easier by the positively charged liposomes encapsulating negatively charged nucleic acids (Xu et al., 2019). The most popular CRISPR/Cas-9 component carriers are polymeric nanoparticles, like polyethyleneimine and poly-L-lysine. Polymer-based nanoparticles can also move through the complex in the membrane through endocytosis, much like lipid nanoparticles can. Viral vectors are the *in vivo* CRISPR/Cas-9 delivery masters by nature (Behr et al., 2021) due to their superior delivery effectiveness compared to physical and chemical approaches, vectors such as adenoviral vectors (AVs), adeno-associated viruses (AAVs), and lentivirus vectors (LVs) are currently being employed extensively as delivery methods. Due to their low immunogenicity and lack of integration into the host cell’s DNA when compared to other viral vectors, AAVs are among them the most often employed vectors. The huge size of the Cas-9 protein and the restricted ability of the virus to clone remain significant issues. One approach to overcome this obstacle is to bundle Cas-9 and sgRNA onto different AAVs, which are then co-transfected into cells. Recent techniques enable the packaging of sgRNA and Cas-9 in the same AAVs by using a smaller strain of Cas-9 from Staphylococcus aureus (SaCas-9) as opposed to the more often utilized SpCas-9 (Horodecka and Düchler, 2021). Extracellular vehicles (EVs) have recently demonstrated significant promise for *in vivo* CRISPR/Cas-9 distribution by avoiding some of the drawbacks of viral and non-viral approaches. The off-target effect, also known as the planned sgRNA will mismatch with the non-target DNA and might lead to nonspecific, unexpected genetic change. The 20-nucleotide sgRNA sequences and the PAM sequences next to the target genome dictate the CRISPR/Cas-9 target efficiency. More than three mismatches between the target sequence and the 20-nucleotide sgRNA have been demonstrated to cause off-target effects (Collias and Beisel, 2021). The off-target impact limits the use of the CRISPR/Cas-9 editing system for therapeutic reasons by potentially causing detrimental events like sequence mutation, deletion, rearrangement, immunological response, and oncogene activation. Several tactics have been devised to reduce the probability of the CRISPR/Cas-9 off-target effect, including the improvement of sgRNA, modification of the Cas-9 nuclease, use of additional Cas-variants, and the use of anti-CRISPR proteins (Pausch et al., 2020; Yılmaz, 2021).

A crucial first step to lessen the off-target effect is to choose and create an appropriate sgRNA for the intended DNA sequence, strategies such as GC content, sgRNA length, and chemical alterations of sgRNA must be taken into account while creating sgRNA. In general, research showed that truncation (short length of sgRNA) and integration of 2-O-methyl-3-phosphonoacetate in the sgRNA ribose phosphate backbone are the recommended strategies to boost the efficacy of CRISPR/Cas-9 genome editing. Another strategy to lessen off-target effects involves altering the Cas-9 protein to enhance its nuclease selectivity. For instance, changing either of the HNH or RuvC catalytic residues in the Cas-9 nuclease will cause Cas9 to change into nickase, which can only cause a single-stranded break rather than a blunt cleavage. The off-target effect can be reduced by 100 to 1500 times by using Cas-9’s inactivated RuvC domain in combination with sgRNA, according to reports. Additionally, the nuclease Cas-12a (formerly called Cpf1) is a type V CRISPR/Cas system that has high genome editing effectiveness. The CRISPR/Cas-12a system, in contrast to the CRISPR/Cas-9 system, can convert precrRNA into mature crRNA without tracrRNA, resulting in a smaller plasmid construct (Yılmaz, 2021). The Cas-12a protein gives more precision at the target gene loci than Cas-9 because it identifies a T-rich (5-TTTN) PAM motif instead of a 5-NGG sequence. With the advent of multicomponent Class, I CRISPR proteins like Cas-3 and Cas-10, genome editing efficiency has improved over Cas-9. The ATP-dependent nuclease/helicase Cas-3 can remove a significant amount of DNA from the target spot without having a noticeable off-target effect. For instance, in induced pluripotent stem cells, the Cas-3-mediated method was used to repair the DMD gene. The Cas-10 protein can recognize sequences even in the presence of point mutations and does not require the PAM sequence. Small proteins called anti-CRISPR (Acr) proteins which are generated from phages, prevent the CRISPR/Cas system from functioning (Liu G et al., 2020, Liu L et al., 2021), and recently found a technique to lessen CRISPR/Cas-9 off-target effects. AcrIIA4 preferentially targets Cas-9 nuclease from Acr proteins. By imitating DNA and attaching it to the Cas-9 site, AcrIIA4 prevents further cleavage in regions outside the target region. The ethics and safety of CRISPR/Cas-9 gene editing have also come under fire globally. Many scientists believe that there is still much work to be done to improve accuracy and ensure that modifications made to one area of the genome do not have unintended implications because the technology is still in its infancy and there is little knowledge about the genome (Anandu and Tanuj, 2020; Ali et al., 2021; Duan et al., 2021; Shah et al., 2021: Zhang et al., 2021; Curtin et al., 2022). As a cutting-edge molecular biology tool, CRISPR/Cas9 genome editing enables us to carry out precise and effective target modification, identify novel opportunities for evolving new plant variations using deletions, insertions, and substitutions, and thereby encourage the expediting of food crop enhancement. To date, rice has successfully benefited from the use of genome-editing techniques utilizing CRISPR/Cas9 for the development of drought tolerance, cold tolerance, salt, and heavy metal sensitivity (Ahmad et al., 2021; Aslam et al., 2021; Wang et al., 2021; Ahmad, 2022; Ashraf et al., 2022; Wang L et al., 2022).

CRISPR/Cas9 recognizes and targets the genetic material of foreign DNA in three steps: adaptation/acquisition, expression, and interference. The recognition, invasion, and binding of donor DNA that has been chopped into short segments and joined within the CRISPR locus are all parts of the adaptation/acquisition phase (**Figure 2**) (Ali et al., 2021; Duan et al., 2021; Zhang et al., 2021). After that, the CRISPR locus is translated to produce crRNA, which instructs endonucleases to target viral components by complementary base pairing. Since the protospacer contains the G-rich base pair (5′- NGG-3′), PAMs are used as recognition motifs to adapt to or acquire the targeted site. In the second phase of the CRISPR/Cas9 execution mechanism, the lengthy Pre- crRNA is purposely transcribed from the CRISPR nucleus and replicated into crRNAs using Cas9 proteins. Recently, it was found that tracrRNA is also processed during Streptococcus pyogenes pre-crRNA synthesis (Aslam et al., 2021; Khan et al., 2021; Ashraf et al., 2022). The tracrRNA is linked to the repeat and the 46-crRNA site through complementary base pairing, which facilitates the conversion of precrRNA into crRNA. The improved crRNAs join the associated antiviral defense complex of the CRISPR system to help locate and separate a specific target area of donor DNA (Nawaz et al., 2019; Naim et al., 2020; Nakamura et al., 2021; Kong et al., 2022; Kumagai et al., 2022). For the sgRNA to be able to direct the cleavage of the Cas9 protein complex from the precise target region, resulting in immunity against pathogen resistance, the approach needs a Cas9 protein at the interference stage (Ahmad, 2022; Ashraf et al., 2022).

The CRISPR/Cas system has become a potent tool for precise genome editing with a wide range of uses, including genetic code rewriting, base editing, knockout, and knock-in. Both Cas9 and Cas12 are among the most frequently employed Cas nucleases for DNA manipulation and are members of the Class 2 CRISPR/Cas system (Khan et al., 2021; Wang Z et al., 2022; Ahmad M., 2022; Ashraf et al., 2022). Although both CRISPRi and CRISPRa could be utilized to control transcription, Cas9 nuclease was unable to target RNA at the post-transcriptional level. The strongest method for posttranscriptional gene controls up until now was RNA interference (RNAi), however, the discovery of Cas13, an RNA-targeting Cas nuclease, offers an alternative to RNAi for precise RNA editing. Cas13 is a single effector Cas protein that is a member of class 2 of the CRISPR/Cas system, like Cas9 and Cas12, instead of relying on PAM, it uses the PFS region to cleave ssRNA. (Ashraf et al., 2022; Wang et al., 2022).

### 3.2 CRISPR/Cas9 system to perform targeted editing

Here, we describe a method for using the CRISPR/Cas9 system to perform targeted editing (**Figure 3**). The first step is choosing a target site to be positioned at the 3′ ends or downstream of the protospacer of the short PAM sequence. Applications of the CRISPR/Cas9 technology on a broad scale must pay close attention to the selection of the target site. An important mutation site can have the fewest/no off-target consequences by selecting the right target site(s). In model organisms, such as rice crops, a wide range of bioinformatics online tools are available for the creation of sgRNA as well as the discovery of off-target (Khan et al., 2021; Ashraf et al., 2022; Chen et al., 2022). Designing the oligonucleotide or primers that are relevant to the target site comes next after the target site has been found. The created primers incorporate a DNA fragment encoding the sgRNA scaffold, which is positioned under the proper promoter to maximize expression. Appropriate adapter sequences should be included for cloning. The forward primer must have 5′-GGCA-3 adaptors, while the inverse primary must have a 5′-AAAC-3′ adaptor (**Figure 3**). The constructs are then inserted into the Cas9 expression vector (pC1300 Cas9) and delivered into the target cell after the protospacer sequence has been subcloned into an SK-sgRNA vector or cassette (an example vector for our case study), using the proper techniques, such as callus bombardment, protoplast transformation, or agrobacterium transformation, cereal plant cells can be related (S. Molla et al., 2021; Kumar et al., 2022; LaManna et al., 2022; Li et al., 2022; Liu et al., 2022a; Liu et al., 2022b)

### 3.3 Important factors to keep in mind when choosing gRNA

For the best possible application of CRISPR technology, the optimal methods for gRNA selection must be developed, carried out, and evaluated. Thus, when creating sgRNAs for use in the CRISPR technique, the optimum sgRNA mostly depends on the goal: gene knockout, base editing, or modification of gene expression.

When creating sgRNAs, the following factors need to be taken into account:

- The essential domains or close to the 5′ ends of the coding area should be where the DSB is first introduced. The 3′ ends of the target sequence must have a PAM sequence (5′-NGG-3), if it is chosen to be close to an adjacent protospacer motif (PAM).
- The Cas9 nuclease will digest about three bases upstream of the PAM structure, hence the target sequence (crRNA) should be upstream of the PAM structure. The PAM sequence is necessary for cleavages; however, it is not a part of the sgRNA sequence and shouldn’t be included in the sgRNA sequence itself because it is not necessary.
- Targets with poly-T and very low or very high GC content (25% or 80%) have low editing efficiency when choosing the correct target sequence for on-target activity. Similar to this, target sites with eight or more contiguous nucleotides have poor editing efficiency and ought to be connected to the sgRNA sequence. Reduced off-target effects are a further factor to be taken into account before the CRISPR experiment begins.
- The position of the gene target is not always crucial, but the gRNA sequence needs to be created in a way that maximizes on-target sites while minimizing off-target sites. The sgRNA/Cas9 nuclease combination may be more likely to target potential off-target locations with higher scores. To prevent off-target editing, the chosen gRNA spacers/target sequence should be sufficiently specific.

It may seem apparent, but when developing a CRISPR/Cas9 vector for an experiment, it is important to maximize on-target activity while limiting off-target activity (Khan et al., 2021; (Molla et al., 2021; Kumar et al., 2022; LaManna et al., 2022; Li et al., 2022; Liu et al., 2022a; Liu et al., 2022b)

### 3.4 Emerging CRISPR/Cas GE tools and why it is more sustainable than previous CRISPR/Cas9

Numerous recent studies have concentrated on altering the CRISPR/Cas9 system to increase its effectiveness and precision. GE currently makes use of new CRISPR/Cas tools (spCas9-NG, base editing, prime editing, xCas9, Cpf1, Cas13, and Cas14). Common Cas9 (SpCas9) recognizes canonical NGG PAM, which restricts the rice genome’s editable space. In numerous research, it has been investigated if additional Cas effectors (such as Cpf1 for AT-rich PAMs) and designed Cas9 variants (such as VQR for NGA PAMs and VRER for NGCG PAMs) may be combined with other PAMs for rice genome editing to overcome this limitation. The effectiveness of xCas9 and SpCas9-NG for gene editing in rice has been evaluated by researchers using stable transgenic lines. In rice containing NG and GAT PAM sequences, xCas9 was demonstrated to effectively induce mutations at target locations (Liu et al., 2020; Khan et al., 2021).

For Cas9 to be recruited, tracrRNA and crRNA are both necessary. The Cas9 then generates a DSB, producing blunt ends, TracrRNA is not necessary for the CRISPR/Cas12a System because Cas12a is a single-component protein that identifies T-rich PAM at the 5′ ends of the target sequence. The DSB produces sticky ends with a 5′ overhang and staggered cuts. The target site is selected using cytidine deaminase combined with dCas9 in the nuclear base-editing system. There is no double-strand break (DSB), cytidine deaminase transforms C straight into U, and a CT substitution can be fixed during mismatch repair when the changed strand is utilized as a template. RNA base editing “A-to-I” editing in the REPAIR system makes use of dCas13 fused to ADAR2, REPAIR uses a 50-nucleotide mRNA-gRNA duplex with 50-nucleotide RNA (Liu et al., 2020; LaManna et al., 2022; Li et al., 2022; Liu G et al., 2022; Liu Q et al., 2022), target A is determined by the “A-C” mismatch in the RNA-gRNA duplex, SAVE the system from “C-to-U” editing a 30-nucleotide spacer added to a gRNA produces the best outcomes. An induced “C-C” or “C-U” mismatch in the mRNA-gRNA duplex identifies the target “C.” Prime editing (E). Reverse transcription is accomplished by:

(a) the fusion protein nicking the desired DNA sequence at the PAM strand;
(b) the exposed 3′ hydroxyl group priming the reverse transcription (RT) of the RT template of the prime editing gRNA (pegRNA);
(c) the branched intermediate form containing two flaps of DNA: a 3′ flap (containing the edited sequence) and a 5′ flap (containing the dispensable, unedited;
(d) ligation and mismatch repair; either incorporating the edited strand or removing it.

Using PCR site-directed mutagenesis, two different xCas9 variants (xCas9 3.6 & xCas9 3.7) were produced. The viability of genome editing with xCas9 variations in rice was examined by making 18 target sites including GAA, GAT, and NG PAMs in three endogenous genes—MOC1, D14, and PDS. The results suggested that rice genome editing with xCas9 could be expanded by using certain previously reported non-canonical PAMs. The vectors pCas9 3.6 (OsU6) and pCas9 3.7 were created by replacing the wild-type SpCas9 in the pCas9 (OsU6) vector with xCas9 3.6 and 3.7, respectively (OsU6). Overall, xCas9 3.7 worked better for genome editing in rice than xCas9 3.6, proving that xCas9 variations have the potential to develop into adaptable tools that will further our understanding of genome editing (Liu et al., 2020).

Recently, it was discovered that SpCas9-NG, a SpCas9 variation rationally created based on the structure of the SpCas9-sgRNA-DNA complex, can identify relaxed NG PAM sequences. When compared to SpCas9, xCas9 and SpCas9-NG both identify PAM sequences that are laxer. SpCas9-NG is more suited for base editing in rice, while xCas9 and SpCas9-NG variants that identify relaxed NG PAMs can function more effectively in rice. The target scope for rice has been greatly widened by the flexible GE tools developed, which is advantageous for basic plant research and crop genetic advancement. Contrary to SpCas9, xCas9 generally exhibits stronger DNA specificity and editing efficiency, less off-target activity, and increased PAM compatibility (Aslam et al., 2021; Khan et al., 2021; Ahmad, 2022; Ashraf et al., 2022). Prime editing enables the targeted induction of insertions (up to 15 nt) and deletions, as shown for rice, and multiple base changes in rice (up to 40nt). Once inside, the fusion protein nicks the cell’s DNA at the target sequence, starting reverse transcription of the template sequence present in the pegRNA. The pegRNA and fusion protein are then introduced to the target cell. When the target cell is exposed to the pegRNA and fusion protein, the fusion protein nicks the cell’s DNA at the target region, triggering the start of reverse transcription of the DNA template found in the pegRNA (Ahmad, 2022; Ashraf et al., 2022) an edited strand of DNA and an unedited strand of DNA are created as a result. To create double-stranded DNA, the unedited strand is first cut, and then the newly edited strand is annealed back together. A recently identified CRISPR effector called Cas13 may specifically target viral RNAs and endogenous RNAs in plant cells. Cas13 is highly efficient and selective at targeting RNA. To restore functioning proteins and stop the progression of the disease, Cas13 has been employed to drive *ADAR2* deaminase in human cells for RNA modification (converting adenosine to inosine) (Ahmad M., 2022; Ashraf et al., 2022). Recent research has revealed the existence of a brand-new class II type VI CRISPR type called CRISPR/Cas13a. The presence of higher eukaryotic and prokaryotic nucleotide-binding (HEPN) domains is what gives it its RNase activity. In addition to the Cas9 proteins, Cas12a (Cpf1) and Cas12b (C2c1) from the class 2 type V CRISPR/Cas system has been repurposed for GE. They differ significantly from Cas9 proteins in several ways, including the recognition of AT-rich PAM sequences by Cas12a and Cas12b as opposed to GC-rich PAM sequences by Cas9 (Khan et al., 2021). A T-rich PAM sequence (5′-TTTN-3′ or 5′-TTTV-3′; V might be A, C, or G) at the end of the protospacer sequence is necessary for *Cpf1*. While Cas9 creates DNA breaks with blunt endpoints, *Cpf1* creates DSBs with staggered ends at the distal position of a PAM, which may offer additional benefits, especially for knock-in techniques, and may boost the efficiency of the process of NHEJ-based gene insertion (Ahmad M., 2022; Ashraf et al., 2022; (LaManna et al., 2022; Li et al., 2022; Liu et al., 2022a; Liu et al., 2022b).

### 3.5 Progress and recent updates in CRISPR-Cas genome editing technology on food security/climate resilience

We might be able to get beyond the limitations of protospacer adjacent motif sequences, target specificities, and Cas9’s high protein size thanks to the new CRISPR-Cas systems. The novel nucleases might assist in lowering licensing costs for breeding businesses, making it simpler to use gene editing to enhance crops (He et al., 2022). Multiplex genome editing is the simultaneous targeting of numerous targets or genes, whether related or unrelated plants have been reported to undergo multiplex genome editing (Li et al., 2019; Xiong et al., 2021). High-specificity guide RNAs are created using some computer programmers, including CRISPR-P, CHOP-CHOP, Benchling, cCTOP, and CRISPR GE, to assist prevent off-target locations. However, off-targets in the genome can be found using software programs like CasOffinder. Utilizing previously created Cas variants is another method for reducing off-target modifications. sgRNAs with a high percentage of GC may encourage off-target mutations (>70%) (Hua et al., 2022; Randall et al., 2022).

LaManna et al. (2022) demonstrated that mutant Cas9’s PAM flexibility results in less effective editing by contrasting it to two recently created PAM-flexible variants (Cas9-NG and xCas9). The authors created the functional hybrid enzyme “xCas9-NG” by fusing Cas9-NG and xCas9, which was superior in both types of transcription activation. By base editing A to G and C to T at A-rich PAMs created iSpyMacCas9, a hybrid Cas9 variant that permits targeted mutagenesis. Sretenovic et al. altered the rice genome in 2021 using a related technique by focusing on the promoter region of the Wx gene, the base editing approach is used in translation to improve low amylose rice cultivars (Xu et al., 2019). The discovery of a CRISPR array and a single 70 kDa protein-Cas, a minimally functional CRISPR/Cas system is a recent development in CRISPR technology (Wang et al., 2021). Large bacteriophages have genomes that contain all of their genetic material contrary to Cas9, Cas targets non-host genetic material using a single active site for CRISPR RNA (crRNA) processing and crRNA-guided DNA breakage. DNA detection and genome editing benefit from the most cutting-edge high-tech equipment due to its low molecular weight, which is half that of Cas9 and Cas12a, the Cas is favorable for cellular dispersion, hence enhancing the collection of genomes editing tools (Puchta et al., 2022). In tomatoes, CRISPR-Cas10 results in minor Indels and bidirectional long-range deletions of up to 7.2 kb (Osakabe et al., 2021). The creation of novel nucleases and their use in genome editing will remain an intriguing area of study since they have the potential to provide a variety of novel outcomes that are both exciting and/or unexpected soon (Wang et al., 2021). Recently particle bombardment was effectively used by Kumagai and colleagues to transport the Cas9-CRISPR RNP complex into the shoot apical meristem of wheat. This method eliminates the time-consuming and labor-intensive process of tissue culture. Additionally, their approach permits the alteration of genes in plants that are resistant to transformation (Kumagai et al., 2022). The next stage is to evaluate the modified events to spot the intended outcomes once the gene editing reagents have performed the genetic adjustments in the cells. However, next-generation sequencing-based tools for determining the effects of gene editing allow for the simultaneous analysis of many target locations in various samples, greatly extending the reach and efficiency of the procedure (Huang et al., 2022). Huawei Zhang and colleagues insert two gRNA units for some species that are resistant to transformation, but haploid induction combined with gene editing may be an effective alternative by using a visible marker can help with editing event analysis and non-chimeric plant selection in one plasmid (He et al., 2022). The letter by XiuFeng Sun and colleagues, which describes the use of a multiplex of gRNAs in rice to apply the “suicide” gene technique, is also quite pertinent to the topic of DNA-free editing (Liu et al., 2022a). They employ Transgene Killer CRISPR technology (He et al., 2018) to induce the spatial-temporal formation of suicide cassettes including the BARNASE and Cytoplasmic Male Sterility 2 (CMS) genes to destroy transgene-expressing cells. The transgene-free rice plants produced with double to sextuple mutations have significantly decreased the generation time needed to accomplish this goal (Kong et al., 2022). Fortunately, point mutations, insertions, and almost every other kind of genomic change can be carried out using CRISPR systems. By creating site-specific double-strand breaks, CRISPR techniques successfully insert targeted changes into genomes (DSBs). However, because plant cells mostly repair DSBs through the non-homologous end-joining (NHEJ) pathway, the ensuing mutations might vary and are challenging to predict. NHEJ-based mutagenesis has this disadvantage, especially if a precise change to the plant genome is required (Osakabe et al., 2020; Wang et al., 2020; Osakabe et al., 2021). Homologous recombination (HR), while being a modest (DSB) repair mechanism in somatic plant cells, provides another technique for creating precise genetic modifications. In the presence of a single-stranded or double-stranded DNA template, HR enables the accurate incorporation of genomic information. GT can also use HR to precisely incorporate genomic modifications ranging from a single base pair (bp) to kilo bps (Xiong et al., 2021; Akella et al., 2022). Akella and colleagues used single-stranded oligo-deoxynucleotides and Cas9 RNPs to perform Chlamydomonas GT (Akella et al., 2022) and claim to have successfully recovered precise mutations in an intriguing gene. They refined the procedure by concurrently targeting a second gene that provides a selectable marker such as protoporphyrinogen IX oxidase or aceto-lactate synthase, they may locate scar-free GT of the target gene. The method could be useful for obtaining GT events from other important genes besides Chlamydomonas. In a study (Kumar et al., 2022) instead of the conventional Agrobacterium, scientists used the soil bacterium Ochrobactrum haywardense to deliver the Cas9 components and the donor template into the soybean embryonic axis. They have plants that have precise heritable targeting events and can regenerate modified T0 plants in under two months. This is a big development for achieving GT in a significant soybean crop in terms of both frequency and the absence of any selection criteria. One of the significant difficulties is the potential off-target effects of cytosine base editors (CBEs), which alter a C to a T. The CBE development is critical for its high efficacy, but it is also essential for testing the method’s specificity however Yiping Qi and colleagues’ *A3A/Y130F-BE3* has been tested for effective C-to-T base editing in tomato (Hu et al., 2021; Randall et al., 2022). Hua et al. (2022) describe a variety of ways utilized by researchers to improve prime editing in plants, albeit their efficacy has been restricted.

One of the most astonishing outcomes of plant gene editing is the *de novo* domestication of new crops from wild relatives thanks to breakthroughs in gene editing, multiple genes can now be modified at the same time to produce mutants in S. peruvianum for 110 genes implicated in the synthesis of small-interfering RNAs and disease resistance for gene editing, they employ both diploid and tetraploid protoplasts produced from shoots grown *in vitro*. They found that gene editing and polyethylene glycol (PEG)-Ca2+-mediated transfection did not affect the ploidy levels of the regenerated plants (Hsu et al., 2022). The recently described transgene-free gene editing and protoplast regeneration technology will allow S. peruvianum to be domesticated, while also significantly accelerating tomato polyploidization. Gene editing enhances the diversity of routes and the stacking of various traits. Plant architecture and grain size are two of the most important agronomically significant characteristics influenced by Brussino steroids (BRs) because some of the BR mutant combinations exhibit various developmental patterns, BR research may benefit and rice architecture may improve. Gene-editing events are most commonly detected in nuclear genes the mitochondrial and chloroplast genomes, on the other hand, are two additional plant genomes that are essential for ordinary plant development and growth focus on several members of three gene families in the BR signaling pathway by multiplexing CRISPR/Cas9-based gene editing (Omukai et al., 2022; Liu et al., 2022b).

**Figure 4:**
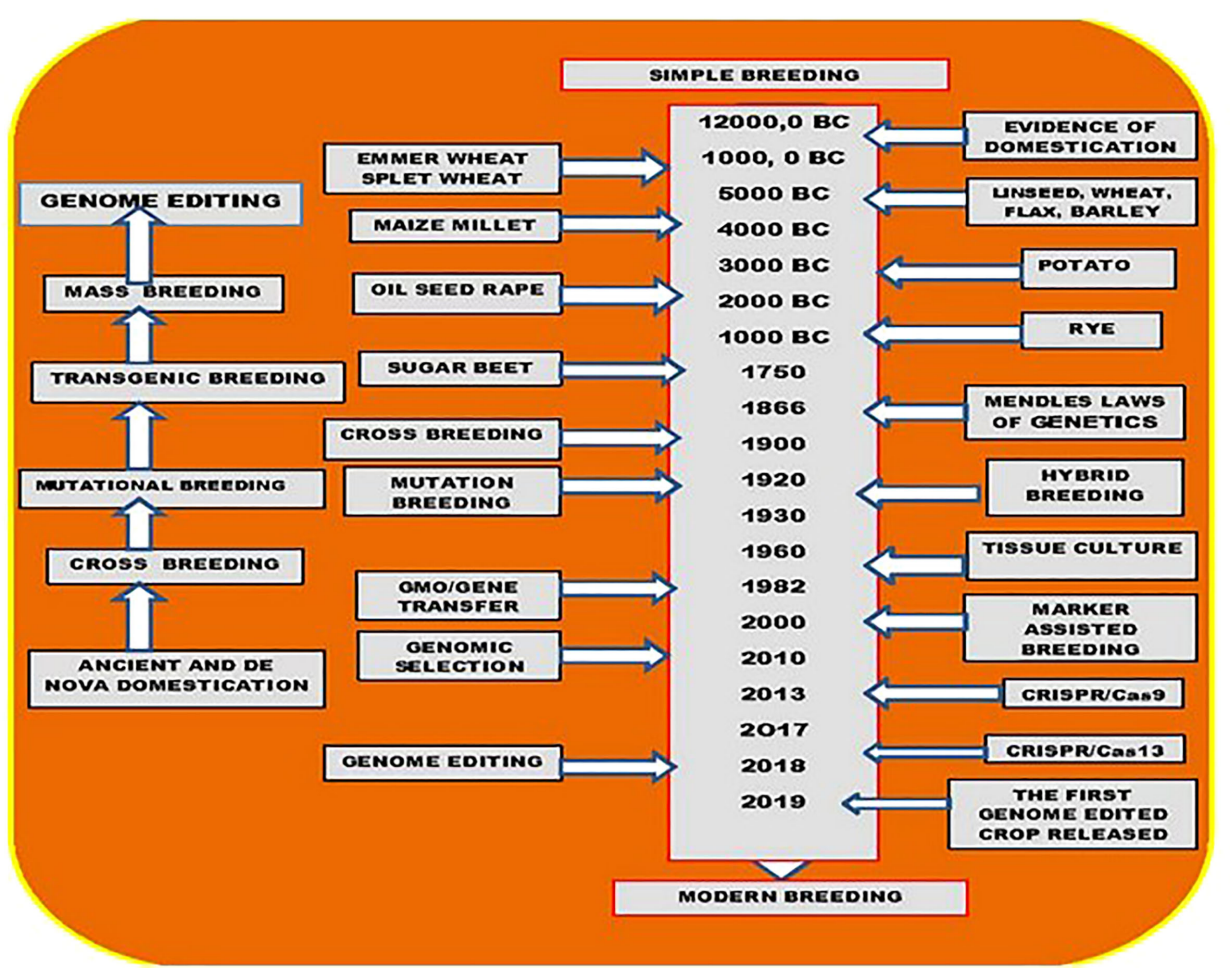
Plant breeding milestones. The start of domestication and initial plant breeding dates back around 12,000 B.C. when the living style of Human-being changed from gathering and hunting to agriculture. The first-ever domesticated plant was emmer wheat. Since then, ancient domestication and selective breeding were dominant until the discovery of Mendel’s laws of genetics. The laws of genetics triggered and enhanced the crossbreeding wave. A milestone in plant breeding that plays an essential role in modern plant breeding was the invention of the totipotency of plant cells in the early 1900s by Gottlieb Haberlandt. As a result, the first *in vitro* tissue culture was introduced in 1960 with carrots. Plant tissue culture was the critical step for generating the first Agrobacterium-mediated transgenic tomato in 1994, known as transgenic breeding. In the meantime, mutational breeding using chemical or physical agents was also introduced in the 1930s and played an important role in generating diverse genetic materials for crop breeding. Biochemical markers further enhanced crossbreeding in marker-assisted selection (MAS) breeding. The recently emerging genome editing approaches have revolutionized plant breeding to precision levels that have never been obtained before. High oleic acid soybean, the first genome-edited crop that was released in 2019, has been opening the wave of genome-edited precision breeding in plants.

Furthermore, altering the genomes of the two organelles offers great promise for enhancing plant breeding. CMS (Cytoplasmic Male sterility) related genes have been discovered in certain plant mitochondrial genomes, enabling the production of high-yielding F1 hybrid agricultural seeds, *Orf352* is one of these genes in rice. One of the earliest amphidiploid crops to be produced by human civilization is Indian mustard (Brassica juncea). This allotetraploid plant species with a total genomic size of 1068 Mb evolved as a result of natural interspecific crossing between the diploid progenitors B. nigra (BB) and B. rapa (AA). The entire genomic sequence of this allotetraploid crop and its diploid ancestors must be decoded to genetically improve the desired features. To understand the structure and functional annotation of genes in the genome, numerous genome and transcriptome sequencing initiatives have been carried out. Similar to this, species-specific molecular insights into key agronomic properties including disease resistance, resilience to climatic perturbations, and fatty acid production were obtained using this genomic data. Thus, the nuclear and organellar genome sequencing work in B. juncea improved our comprehension of the intricate allotetraploid architecture and laid the groundwork for future use of the data in translational genomics and precession breeding. Their findings suggest that *orf352* is required for CMS and that the amino acids 179 to 210 from *orf352* may aid in pollen abortion (Ramkumar et al., 2022). Pal Maliga and colleagues after a decade successfully used a multiplexing CRISPR system comprised of four gRNAs and egg-cell-specific Cas9 expression to create transgenic B. napus seedlings with dysfunctional *ACC2* genes, they exhibit a spectinomycin hypersensitive phenotype, it is hoped that these mutant plants will be appropriate for chloroplast transformation in significant foods like rice, wheat, tomato, banana, citrus, grapes, cassava, and cucumber have all shown signs of pathogen resistance when they were grown on CRISPR-engineered plants (**Table 1**) (LaManna et al., 2022; Li et al., 2022; Liu et al., 2022a; Liu et al., 2022b). Since these loci confer resistance to numerous pathogen species or breeds, broad-spectrum resistance is an efficient disease control method for plants. Crop genomes have been quickly modified using CRISPR to create wide mutants that are resistant to environmental stresses. Many unique mutants of rice with high yield and exceptional storability were created as a result of the simultaneous editing of three genes using the CRISPR-Cas9 system: OsPIN5b (a gene for pods per plant), GS3 (a gene for particle sizes), and OsMYB30 (a gene for cold stress). They are also stable in the T2 phase (Zeng et al., 2019; Chen et al., 2022). Under the guidance of Feronia (Fer) conserved regions, BZR (brassinosteroids regulator) overexpression provided tomatoes with thermal sensitivity. Important crops like rice, wheat, tomatoes, brassica, and others have demonstrated good stress tolerance by altering key transcription factors (Li et al., 2019). In ABA signaling, *ABF2* is an essential transcription factor. According to the Bhat et al. review, significant research has been done on rice’s tolerance to salinity (2021). By providing an off-switch mechanism, the Arabidopsis PARAQUAT TOLERANCE 3 (*AtPQT3*) gene, which codes for an E3 ubiquitin ligase, enables plants to balance their responses to stress and growth. Using CRISPR-Cas9, a rice homolog of *AtPQT3* was removed the resulting OsPQT3 knockout mutants (*ospqt3*) showed improved agronomic performance, superior yield under salt stress in both greenhouse and field conditions, and greater resilience to oxidative and salt stress in compared to the wild type. To add desirable traits to four stress-tolerant wild-tomato accessions, edited multiplex CRISPR-Cas9 genes involved in morphology, flower and fruit development, and ascorbic acid production. Domesticated phenotypes and parental traits for disease resistance and salt tolerance were present in the Cas9-free progeny of changed plants (He et al., 2022). CRISPR-Cas9-mediated gene editing of the GS3 and Gn1a genes, which regulate grain size and number, resulted in the production of three mutant genotypes (*gs3-N9108, gs3-Z22*, and *gs3gn1a-Z22*) in rice with 3-7% higher grain yields than the WT. The genome-edited mutants created by altering the *GL2/OsGRF4* and *OsGRF3* genes, which are in charge of grain yield and size, respectively, had larger grains (Hsu et al., 2022). The number of grains, the size of the grains, and the number of tillers in the T2 generation were all increased as a result of CRISPR-Cas9 editing of the genes *Gn1a, DEP1, GS3*, IPA1, and other genes (He et al., 2020; Hu et al., 2021; He et al., 2022). Breeders’ major goal is to biofortify grains to make them more nutrient-dense and to ward off diseases caused by nutrient deficiency. Rice lysine content has risen 25 times as a result of editing the genes for the crucial lysine biosynthesis enzymes AK (less) and DHPS (dap) while maintaining the starch composition of these high-lysine lines also demonstrated improved physical and chemical properties. The plants grew normally in field experiments, with just slight differences in plant height and grain color. High quantities of beta-carotene are produced by rice that has undergone marker-free gene editing of the *CrtI* and *PSY* genes. Several nutrients, such as -aminobutyric acid, were employed to produce biofortified tomatoes (GABA) (Hu et al., 2021). GABA is a neurotransmitter that controls anxiety and blood pressure. The deletion of the C-terminal autoinhibitory region of glutamate decarboxylase, a key enzyme in the synthesis of GABA, results in mutant tomatoes with a sevenfold increase in GABA accumulation. By using CRISPR-Cas9 to modify BnTT8 homologs, it was possible to create rapeseed mutants with yellow seeds while increasing the oil content in the T2 generation by 9.47%. Additionally, studies on Cas9-mediated starch mutagenesis were conducted (Yin et al., 2018; Xiong et al., 2021). In tetraploid potatoes, branching enzymes (SBEs) increased the amount of resistant starch and produced transgene-free mutant potato lines, which may help insulin better control blood sugar levels (Tran et al., 2020; Tran et al., 2020; Oz et al., 2021; Teferra, 2021). CRISPR/Cas9-mediated ITPK gene knock-out in rapeseed reduced phytic acid content by 35% without affecting plant performance. By employing the CRISPR/Cas9 technology to target a conserved section of the -gliadin genes, low-gluten wheat lines without transgenes have been produced. One notable use of the CRISPR/Cas9 technique in rice breeding is the development of cultivars of rice that are resistant to heavy metal pollution (Oz et al., 2021). The human cancer-causing substance cadmium (Cd) can cause renal failure by modifying the OsNramp5 gene, which regulates Cd uptake by the roots, scientists were able to produce distinctive Indica rice varieties with less Cd buildup in the grain. Evaluations of the Osnramp5 mutants’ performance in the field revealed that higher Cd levels had no impact on either their agronomic characteristics or grain yield. The altering climate continues to be the main obstacle to crop improvement, Therefore, increasing crop productivity in less-than-ideal conditions is the breeders’ top priority discovered the numerous factors affecting crop yield and provided breeding choices for increasing crop output in less-than-ideal circumstances, according to (Tang et al., 2019; Liu L et al., 2020). Genome editing is very crucial for comprehending how genes function during stress reactions and how plants have developed adaptive mechanisms to deal with difficult environmental conditions. The areas where high-yielding and climate-resilient crops can be grown from domestication to the genome editing era are depicted in (**Figure 4**) (Tang et al., 2019; Fernie and Yan, 2019; Voss-Fels et al., 2019; Liu et al., 2021; Rönspies et al., 2021). A range of herbicide resistance will be produced in plants that are essential for ergonomics by selectively selecting the target genes (Hsu et al., 2022). However, the EPSPS, HPPD, and ALS genes from Zea maize, Avena sativa, and Nicotiana tabacum can be modified using CRISPR-Cas technology. Even though most commercially grown plants have bacterial genes transferred into them, researchers were able to enhance the flavor of modern beer by locating and changing a gene that greatly affects its flavor and other alcoholic beverages (Han and Kim, 2019; Hsu et al., 2022). Only if food production is dispersed and increased sustainably, along with extreme poverty, is a world without hunger possible. Most underprivileged and impoverished people worldwide reside in rural parts of developing nations, where they rely on agriculture for food, income, and jobs. International data demonstrate a strong correlation between high rates of undernourishment and low agricultural productivity. Global studies have also demonstrated that raising the earnings of smallholder farmers is the only way to significantly reduce extreme poverty. Therefore, socioeconomic growth depends on continuing increases in agricultural output, and novel plant breeding technologies (NPBTs), such as genome editing, will be able to make a significant contribution to global food security with judicious application and scientifically informed management (Zaidi et al., 2019; Rosenzweig et al., 2020; Tsveta et al., 2021; LaManna et al., 2022; Liu et al., 2020; Chen et al., 2021; Tran et al., 2021; Li et al., 2022; Liu J et al., 2022a; Liu D et al., 2022b).

**Table 1:** Using genome editing CRSPR-Cas to from last four years to increase the ability of major food crops to withstand climate adversity.

Plant Species	Target Genes	Gene Function	Phenotype	Mode of Application	References
**Rice**	*OsPRPl*	Proline-rich protein	Cold sensitive	Mutants exhibited sensitive phenotype after treatment at 6°C for 3 days.	Nawaz et al., 2019
	*OsMYB30*	Transcription factor	Cold tolerance, increased panicle length, enlarged grain size	Mutants exhibited tolerance phenotype after treatment at 4°C for 5-10 days.	Zeng et al., 2019
	*OsAnn5*	Annexin	Cold tolerance	Mutants exhibited tolerance phenotype after treatment at 4-6° C for 3 days.	Shen et al., 2020
	*OsERA1*	ABA signaling and the dehydration response	Enhanced response to drought stress through stomatal regulation	Mutants showed drought-tolerant phenotype under the 8-day watering-off treatment.	Ogata et al., 2020
	*OsSRL1,2*	Regulation of leaf rolling	Enhanced drought tolerance and ABA level	Mutants showed drought-tolerant phenotype under 30-day water-deficient treatment.	Liao et al., 2019
	*OsNAC006*	NAC transcription factor	Heat sensitive	Mutants exhibited sensitive phenotype after treatment at 42° C for 4 days.	Wang et al., 2020
	*OsPUB67*	U-box E3 ubiquitin ligase	Reduced drought tolerance	Mutants showed drought-sensitive phenotype after 10-day no water treatment at tillering stage.	Qin et al., 2020
	*OsRR22*	Involved in both cytokinin signal transduction and metabolism	Enhanced salinity tolerance	Mutants showed salinity-tolerant phenotype under concentrations of 0.75% NaCl solution treatment.	Zhang et al., 2019
	*OsVDE*	Key enzyme of xanthophyll cycle	Enhanced salinity tolerance, reduced water loss	Mutants showed salinity-tolerant phenotype at 100 mM NaCl application.	Wang et al., 2021
	*OsDST*	Drought and salt tolerance gene	Enhanced salinity tolerance, showed significantly broader leaf width and enhanced leaf area	Mutants showed salinity-tolerant phenotype at 200 mM NaCl application.	Santosh Kumar et al., 2020
	*OsNAC041*	NAC transcription factor	Reduced salinity tolerance, enhanced MDA content	Mutants showed salinity-sensitive phenotype at 150 mM NaCl application.	Bo et al., 2019
**Tomato**	*SlIAA9*	Transcriptional regulator for auxin response	Parthenocarpy	Mutants exhibited parthenocarpy phenotype.	Kashojiya et al., 2022
**Tomato**	*SlCPK28*	Protein kinase, Ca^2^+ sensing	Heat sensitive, accumulation of ROS	Mutants exhibited sensitive phenotype and higher H_2_O_2_ content after treatment at 45°C for 12 h.	Hu et al., 2021
	*SlMAPK3*	MAP kinase upregulating HSPs’/HSFs’ genes’ expression	Heat tolerance, reduction of ROS accumulation	Mutants exhibited tolerance phenotype and lower H_2_O_2_ and O2*^—^ contents after treatment at 42°C for 1 day.	Yu et al., 2021
	*SIBZR1*	Transcription factor for brassinosteroid response	Heat tolerance	Mutants exhibited tolerance phenotype after treatment at 42°C/38°C (day/night) for 1 day.	Yu et al., 2019
	*SlNPR1*	A special receptor of salicylic acid	Reduced drought tolerance, increased stomatal aperture	Mutants showed drought-sensitive phenotype without watering for 6 consecutive days.	Li et al., 2019
	*SlLBD40*	Plant-specific transcription factors	Enhanced drought tolerance and reduced stomatal conductance	Mutants showed drought-tolerant phenotype under the 10-day watering cessation treatment.	Liu L et al., 2020
	*SlARF4*	Auxin response factors	Enhanced drought tolerance and stem thickness	Mutants showed drought-tolerant phenotype under the 12-day watering-off treatment.	Chen S et al., 2021
	*SlHyPRP1*	A subgroup of putative plant cell wall glycoproteins	Enhanced salinity tolerance and stem length	Mutants showed salinity-tolerant phenotype at 100 mM and150 mM NaCl application.	Tran et al., 2020
	*SlARF4*	Auxin response factor	Enhanced salinity tolerance, delayed flowering, increased height and leaf curling	Mutants showed salinity-tolerant phenotype at 250 mM NaCl application.	Tran et al., 2020
**Arabidopsis**	*AtAITR* family	ABA-induced transcription repressors	Enhanced drought and salt tolerance, reduced ABA sensitivity	Mutants showed drought-tolerant phenotype after 12-day watering off treatment and 2 days of rewatering.	Chen S et al., 2021
	*AREB1*	ABA-responsive element-binding protein	Enhanced drought tolerance and chlorophyll content	Mutants showed drought-tolerant phenotype under 20% humidity treatment or 20-day cessation of watering.	
	*AtAITR*	ABA-induced transcription repressors	Enhanced salinity tolerance, reduced ABA sensitivity	Mutants showed salinity-tolerant phenotype at 150 mM NaCl application.	
	*ACQOS*	A toll-interleukinl receptor-nucleotidebinding leucine-rich repeat class protein	Enhanced salinity tolerance and chlorophyll content	Mutants showed salinity-tolerant phenotype at 250 mM NaCl application.	
**Soybean**	*GmMYB118*	MYB transcription factor family	Reduced drought and salinity tolerance	Mutants showed drought-sensitive phenotype after 14-day no water treatment.	
	*GmAITR*	ABA-induced Transcription Repressors	Enhanced salinity tolerance, more sensitivity to ABA	Mutants showed salinity-tolerant phenotype at 200 mM NaCl	Wang et al., 2021
**Wheat**	*TaHAG1*	Histone acetyltransferase	Reduced salinity tolerance, more chlorotic leaves and higher Na+ content in the mutants	Mutants showed salinity-sensitive phenotype at 200 mM NaCl application.	Zheng et al., 2021
**Potato**	*Coilin*	A main structural protein controlling the formation, composition, and activity of subnuclear Cajal bodies	Enhanced salinity tolerance, slower yellowing and leaf fall	Mutants showed salinity-tolerant phenotype at 300 mM NaCl application.	

## 4 What benefits might CRISPR/Cas-based gene editing offer

A new study suggests that Cas9-CRISPR gene editing can lead to genomic instability and cell toxicity. Important regions of the genome where gene editing may have unintended effects have been identified by researchers, and these findings have led to recommendations (Science news September 2022). The perfect agricultural plant, according to Karlsruhe Institute of Technology KIT researchers using CRISPR/Cas to suppress chromosomes and prevent genetic exchange, is edible and highly producing while also being disease and insect resistant. However, some of these beneficial traits can be lost during breeding if the pertinent genes are spread out over a large portion of a chromosome. To ensure that advantageous traits can be passed on in tandem, scientists used CRISPR/Cas molecular scissors to invert and so genetically disable nine-tenths of a chromosome, and the traits contained on this chromosome can be passed down intact and become “invisible” to genetic exchange in this way future agricultural and plant cultivation will be more effective and resilient thanks to this technology. For all of the traits on a chromosome to be passed on as a unit, Puchta continues, “we can almost entirely shut down a chromosome, rendering it invisible.” Up until now, for a plant’s traits to be passed down jointly, their corresponding genes are required to be close to one another on the same chromosome. Such genes typically become separated during inheritance if they are dispersed throughout a chromosome, which causes the loss of a desired trait during breeding. The perfect agricultural plant has a high yield, is palatable, and is pest- and disease-resistant (Chen M et al., 2021, Chen S et al., 2021; Ganie et al., 2021; Gardiner et al., 2022; Gehrke et al., 2022)

Editing regulatory elements or splicing sites with CRISPR-Cas nucleases and base editors can regulate transcription activators, repressors, DNA methyl-transferases, de-methylases, and other regulators can be drawn to the promoter region of a gene by employing dead Cas proteins to regulate transcription (Son and Park, 2022). The way forward, future discussions, and challenges to the usage of GMO/LMO products as food or feed are still up for debate. The current trend is to prevent any harm to the environment or any health effects on customers. Cross-kingdom genetic elements are incorporated into plant genomes and overexpressed throughout plant growth and development, which is one of the main justifications for GMO regulation (Teferra, 2021). GE plants, on the other hand, have modified plant genomes that do not include any foreign genetic material, which can sometimes render Indel mutations indistinguishable from wild plants (Son and Park, 2022). GE plants and their derivatives are permitted as food and feed if they are generated through normal breeding and do not include foreign genetic components (El-Mounadi et al., 2020; Menz et al., 2020: Son and Park, 2022). However, because GE varieties can quickly domesticate desirable alleles into superior cultivars at a minimal cost and without linkage drag, they provide hope for preserving and securing world food production to meet demand by 2050. At high herbicide resistance dosages, endogenous promoter-driven expression levels could not be sufficient to maintain enzyme function, instead, the endogenous promoter can be strengthened using the CRISPR-Cas system, potentially enhancing technology for such features (Jin et al., 2021; Son and Park, 2022). Due to the absence of the expensive regulatory process that applies to GMO products, the commercialization of GE products is substantially less expensive than that of GMO products. Small businesses like Calyx and Sentech Seed Co., Ltd. have created innovative biotech goods as a result. There have been several requests for the deregulation of GE processes, which essentially involve cutting the genome with molecular scissors (https://www.aphis.USDA.gov/aphis/ourcus/biotechchannelogy/am-i-regular). This information spurs the creation of new GE crops by other small-scale labs and businesses, supporting the notion that there will be enough food on the planet to feed everyone by the year 2050 (Ahmad et al., 2021: Ali et al., 2021; Arya et al., 2021; Ali and Mahfouz, 2022). The following year, a leafy green vegetable with improved flavor is anticipated to hit the US market as Pair Wise’s first gene-edited product. Next year is anticipated to see the release of other base-edited crops that are now under development. While this was edited using standard CRISPR, other base-edited alterations were also made. One of these is a cherry without stones, which is presently being tested. Cherry trees need more care than row crops do, thus they are now in the development phase and won’t be available for some time. As long as the organisms do not include foreign DNA, certain regulators, like the US Department of Agriculture and the Chinese Ministry of Agriculture, have given CRISPR-edited plants more latitude than transgenic GMOs. But if the public is not persuaded, the technology might disappear. “Success might pave the door for some potentially revolutionary uses, such as lowering the effects of global agriculture on climate change,” the scientific community has concluded. “If you want people to adopt genome-edited crops or food, you must make it more appealing to the public.” however, great potential in enhancing the agronomic effectiveness and flavor of vigorous wild crops to domesticate them. By altering five genes relevant to qualities including fruit size, yield, and nutrient content, the Menz team domesticated a wild South American tomato in 2018. It usually takes 8,000 years to perform this process, and “it’s been 1.5 years now” (El-Mounadi et al., 2020; Menz et al., 2020; Ali et al., 2021; Arya et al., 2021; Ali and Mahfouz, 2022; Son and Park, 2022);. Future efforts to get over these restrictions are probably going to expand the experimental freedom and usefulness of the Cas9-CRISPR toolkit. Precision genetic engineering to increase plant output, nutrition, and climate resilience has been sped up by the discovery and reuse of CRISPR/Cas. New Cas variations have made it easier for molecular biologists to comprehend and control the tightly controlled information flow across (DNA, RNA, and protein), beyond (epigenome and metabolome), and outside the core dogma. But current methods for delivering and carrying out CRISPR-mediated editing in plants are expensive, resource-intensive, time-consuming, and have drawbacks like low efficiency, tissue damage, a small species range, and a constrained capacity for cargo delivery. Recent research on nano- and peptide-carriers (NC and PC) to carry biomolecules (DNA/RNA/proteins/ribonucleoproteins) in plants demonstrates the potential to overcome the issues with conventional procedures and increase the genetic transformation process. To become a viable alternative to current genetic transformation techniques, the field of NC- and PC-assisted delivery of biomolecules is still in its infancy and needs coordinated efforts at the intersection of nanotechnology, proteomics, and plant transgenics. The developments in NC, PC, and NC-PC conjugates make them particularly appealing for delivering biomolecules to plants (Arya et al., 2020; Yip, 2020; Arya et al., 2021; Chen M et al., 2021, Chen S et al., 2021; Ganie et al., 2021; Osakabe et al., 2021; Qin et al., 2020; Tsveta et al., 2021; Zheng et al., 2021; Gardiner et al., 2022; Gehrke et al., 2022; Huang et al., 2022; Ramkumar et al., 2022; Takahashi et al., 2022; Tanwar et al., 2022).

### 4.1 The advantages of CRISPR/Cas9 and the drawbacks of CRISPR/Cas variations

The CRISPR/Cas9 system cannot be packed into viral vectors to be delivered to somatic tissues because of its huge size, which restricts the editing effectiveness of the system. GE needs a smaller-sized CRISPR/Cas to be effective. SpCas9 only identifies the 5′-NGG-3′ PAM sequence close to a 20 nt DNA target site, which reduces its efficacy in comparison to emerging CRISPR/Cas variants. While SpRYCas9, a more recent version, is practically PAM-less, NG-Cas9 is more active. The targeting range of CRISPR-based tools in plant genome engineering is substantially widened by SpRY’s broad PAM compatibility. There is a chance that CRISPR/Cas9 will introduce a lot of off-target mutations into the genome. However, new CRISPR/Cas variants have improved the editing efficiency (fewer off-target mutations) of target bases in the sequence of interest by identifying different PAMs. CRISPR/Cas9 generates mutations at non-specific loci that are homologous to target sites. CRISPR/Cas9-made mutant plants *via* Agrobacterium-mediated transformation systems are more expensive, time-consuming, and resource-intensive (Arya et al., 2021; Ali and Mahfouz, 2022). The commercialization of transgenic crops expressing CRISPR/Cas9 faces challenges in many countries, primarily due to development costs and restrictions imposed by regulatory systems for the field release of genetically modified organisms. On the other hand, the use of tissue culture-free genome editing systems has the potential to improve efficiency.

Using GE technology promises crops with high yields, good quality, and enhanced disease resistance. However, given the negative public impressions about GMOs, people are first cautious to adopt these plants. People do not accept GE plants because they cannot tell the difference between GMO and GE plants. GE plants alter plant traits by adding minute adjustments like deletions, insertions, and targeted mutations using CRISPR/Cas. These GE plants have greatly enhanced their agronomic characteristics. The mutations produced by GE plants do not include any traces of foreign DNA. Additionally, since the CRISPR/Cas system’s guide, RNA (gDNA) is not rDNA, GM and GE are fundamentally different from each other (recombinant DNA). The ability of CRISPR/Cas to produce transgene-free plants with the necessary agronomic traits without adding any foreign DNA has been widely reported in recent years, exempting them from the classification and regulation of GMOs. Recent advances in CRISPR/Cas technology have enabled the development of transgene-free plants that spontaneously acquire the necessary agronomic traits, therefore exempt from the definition of and regulations governing GMOs.

## 5 Conclusions remarks

The creation of fresh instruments and techniques is crucial for the progression of research because it depends on new methods, findings, and theories. The ability of CRISPR screens to identify and investigate gene regulation networks at a pace and scale never before achieved is also very promising. The application of CRISPR screens will significantly progress plant functional and synthetic biology and provide new opportunities for genome analysis at higher resolution. Researchers have improved these key productivity attributes by using CRISPR-Cas9 to alter orthologs of tomato domestication and improve genes that affect plant architecture, flower production, and fruit size in the orphan Solanaceae crop known as “groundcherry” (Physalis pruinose). As a result, applying knowledge from model crops enables the quick development of innovative breeding germplasms and targeted allelic diversity in orphan crops that are not closely related. More crucially, encouraging integrated ecological (biodiversity resources) genomic research has the potential for improved knowledge of antagonistic co-evolutionary interactions as well as more effective use of resistance phenotypes in breeding. Gene prediction may also be used in this situation in addition to pre-breeding efforts and input on restoration optimization. As genome-editing tools have advanced, plant breeding has changed. To overcome obstacles and produce food sustainably, crop breeding and genetics must continually innovate. Cutting-edge molecular biology method CRISPR has already improved our knowledge of how the genome is organized and regulated in living cells from many biological kingdoms. Not only is agriculture being revolutionized by CRISPR, but also industry, the environment, medicine, and other professions. The majority of research employing the CRISPR/Cas9 genome-editing technology has been groundwork/preliminary up to this point due to the relative specificity of each nuclease platform. Future research on the target recognition stringency of each system should make advantage of high-throughput techniques that enable the thorough profiling of off-target cleavage sites. The platform must therefore advance further to be fully utilized, which will boost on-target effectiveness. It has been possible to get around this restriction by employing a variety of techniques, such as altering Cas9 to recognize various PAM sequences. To increase the potential for genome editing in rice, the xCas9 3.7 variant was developed. Additionally, a novel Cas9 variant called SpCas9 was developed that could identify NG PAMs in rice. The fact that many more of these variants are useless in plants emphasizes the need to create new Cas9 variants that can recognize a variety of PAMs. It is also possible to get around the restriction of PAM specificity by using the recently discovered Cas14a system, which does not need PAM but can only target ssDNA. Therefore, a promising strategy for future rice crop enhancement is the quick advancement of research into epigenetic genome alterations in rice. As CRISPR technologies’ reach and potency increase, social and ethical questions concerning its application intensify, and the applications of these potent tools merit further consideration. To build public trust and create regulatory frameworks to control the application of the CRISPR system in agriculture, researchers cannot ignore the difficulties of explaining CRISPR breeding procedures. Whatever obstacles still need to be overcome, the recently developed CRISPR techniques are just the beginning with the potential to provide agriculture with a sustainable future comes the duty to allay public and scientific concerns surrounding the use of these potent new plant breeding technologies.

## Author contributions

The author confirms being the sole contributor of this work and has approved it for publication.
